# Impact of pre-operative cervical spine function on post-operative functional rehabilitation in patients after laminoplasty

**DOI:** 10.3389/fneur.2025.1706836

**Published:** 2025-11-26

**Authors:** Zhiliang Zhang, Yi Zhao, Hu Zhao, Mouwang Zhou, Yanyan Yang, Zhiqian Luo, Junhao Tong, Guoqing Cui, Tao Li, Feifei Zhou

**Affiliations:** 1 Department of Rehabilitation, Peking University Third Hospital, Peking University, Beijing, China; 2 Department of Orthopaedics, Peking University Third Hospital, Peking University, Beijing, China; 3 Department of Rehabilitation, Peking University People's Hospital, Peking University, Beijing, China

**Keywords:** degenerative cervical myelopathy, laminoplasty, posterior cervical muscles, functional rehabilitation, axial symptoms

## Abstract

**Background:**

Laminoplasty has been widely used for the treatment of multilevel degenerative cervical myelopathy (DCM). However, patients often experience persistent axial symptoms, which affect post-operative functional rehabilitation. This study aims to explore the intrinsic connection between the pre-operative structural and functional status of the cervical spine and the post-operative functional rehabilitation of patients undergoing laminoplasty, providing a theoretical basis for individualized rehabilitation strategies for specific populations.

**Methods:**

A retrospective analysis was conducted on the clinical data of patients who underwent laminoplasty and received inpatient rehabilitation treatment. Pre-operative and 3-month post-operative visual analog scale (VAS) score, modified Japanese orthopedic association (mJOA) score, and neck disability index (NDI) score were collected, and correlation analyses were performed with pre-operative cervical flexion and extension range of motion (ROM), cervical muscle strength, and the functional cross-sectional area (fCSA) of posterior cervical muscles measured by magnetic resonance imaging (MRI).

**Results:**

Among the 30 included patients, 17 were males and 13 were females with an average age of 59.70 ± 8.41 years, the follow-up period is 3 months. Correlation analysis revealed that pre-operative cervical extension muscle strength was weakly positively correlated with the post-operative VAS score (*r* = 0.364, *p* = 0.048) and was moderately positively correlated with the NDI score (*r* = 0.448, *p* = 0.013). The regression analysis results showed that extension strength had a significant and positive independent predictive effect on post-operative VAS score (β = 0.256, *p* = 0.025) and NDI score (β = 0.789, *p* = 0.024). For the sum of posterior cervical muscles, pre-operative fCSA of the right multifidus (MF) was weakly negatively correlated with the post-operative NDI score (*r* = −0.369, *p* = 0.045).

**Conclusion:**

Our result suggested that stronger pre-operative cervical extension strength may be important predictors of post-operative functional rehabilitation in patients after laminoplasty, especially in terms of axial symptoms.

## 1 Introduction

Degenerative cervical myelopathy (DCM) is a variety of age-related and genetically associated pathologies, including cervical spondylotic myelopathy, degenerative disc disease, and ligamentous aberrations such as ossification of the posterior longitudinal ligament (1). It is understood that DCM is the most common cause of non-traumatic spinal cord impairment worldwide, literatures reported that the incidence were estimated to occur in 24.2% of asymptomatic/subclinical adults (2, 3). The most consistent feature of DCM is the progression of symptoms rather than the symptoms present. The most common presenting symptom is paraesthesia in the hands which can easily be misdiagnosed. Late-stage presentation of DCM includes motor loss, paralysis and loss of sphincter control (4). Given the natural history of a stepwise deterioration, treatment strategy favors surgical intervention (5), with 1.6 per 100,000 people requiring surgery in their lifetime (6).

Laminoplasty is a posterior decompressive surgery and has been widely used for the treatment of multilevel DCM (7). The long-term clinical follow-up results show that the patients' neurological functions can achieve a relatively ideal recovery after laminoplasty (8, 9), however, post-operative complications such as axial symptoms, C5 palsy and cervical kyphosis seriously affecting the overall efficacy and the rehabilitation process (10–12). To address this surgical dilemma, previous studies have reported modified surgical techniques that maximally preserve the spinal-muscle-ligament stabilizing structures (13–15). In particular, persistent axial pain can be a major cause of dissatisfaction after surgery, even in patients with excellent neurological recovery (16). Therefore, people have come to realize the significance of post-operative rehabilitation treatment for cervical spine function. Some researchers, including those from our research team, have reported the rehabilitation effects of early cervical range of motion (ROM) and resistance muscle strength training for patients after laminoplasty (17–19).

Advancements in clinical decision-making, surgical techniques, and perioperative rehabilitation have allowed doctors to manage increasingly complex cervical spine cases. Continued teamwork is required on the part of orthopedics, rehabilitation physicians, rehabilitation therapists, and nurses to take this access forward. Compared to surgical techniques, the post-operative rehabilitation treatment for cervical spine function has developed slowly, indicating that there are still many overlooked treatment details that we have not addressed. In previous studies, we adopt a team approach for early rehabilitation in patients after laminoplasty, and demonstrated its efficacy in improving quality of life in specific populations (18). However, it still failed to solve the problem that we are most concerned about, namely the therapeutic effect on post-operative axial symptoms. Naghdi et al. (20) discovered that morphological characteristics of pre-operative posterior cervical muscles might be important predictors of functional recovery and post-surgical outcomes in patients with DCM. Therefore, we conducted a retrospective analysis of the clinical data of the patients, attempting to identify the intrinsic connection between pre-operative structural-functional status of cervical spine and post-operative function rehabilitation in patients after laminoplasty. We hypothesized that pre-operative cervical spine function will be associated with post-operative axial symptoms and functional scores. The study aimed to discover potential intervention timing and treatment methods, in order to provide theoretical basis for individualized rehabilitation strategies for specific populations.

## 2 Methods

### 2.1 Study design and participants

We conducted a single-center retrospective study at Peking University Third Hospital, involving 30 patients who underwent laminoplasty and received inpatient rehabilitation treatment after surgery from February 2018 to February 2020. The follow-up period is 3 months. Inclusion criteria: (1) a clear diagnosis of DCM based on medical history, symptoms, signs, and imaging findings; (2) patients who underwent open-door laminoplasty preservation of unilateral (right side) musculo-ligamentous complex by the same surgeon at Peking University Third Hospital (21); (3) age ≥18 years; (4) patients completed a 7-day inpatient rehabilitation treatment from the third day after surgery; (5) complete clinical and follow-up data. Exclusion criteria: (1) Patients with major organic lesions, including cardiac and pulmonary dysfunction, tumors, stroke, Parkinson's disease, etc.; (2) patients who underwent secondary cervical spine surgery during the follow-up period; (3) patients with fractures and severe osteoporosis.

### 2.2 Sample size determination

This study was a exploratory, single-center retrospective study. During the research design phase, we discovered that for patients who undergone laminoplasty, few studies have explored the relationship between cervical pre-operative function and post-rehabilitative function. So there was a lack of previous research data to estimate the effect size for conducting *a priori* sample size calculation. Therefore, we adopted a feasibility sample size design and included all consecutive cases that met all inclusion and exclusion criteria during the study period from February 2018 to February 2020, a total of 30 cases.

### 2.3 Rehabilitation during hospitalization

**Figure 1:**
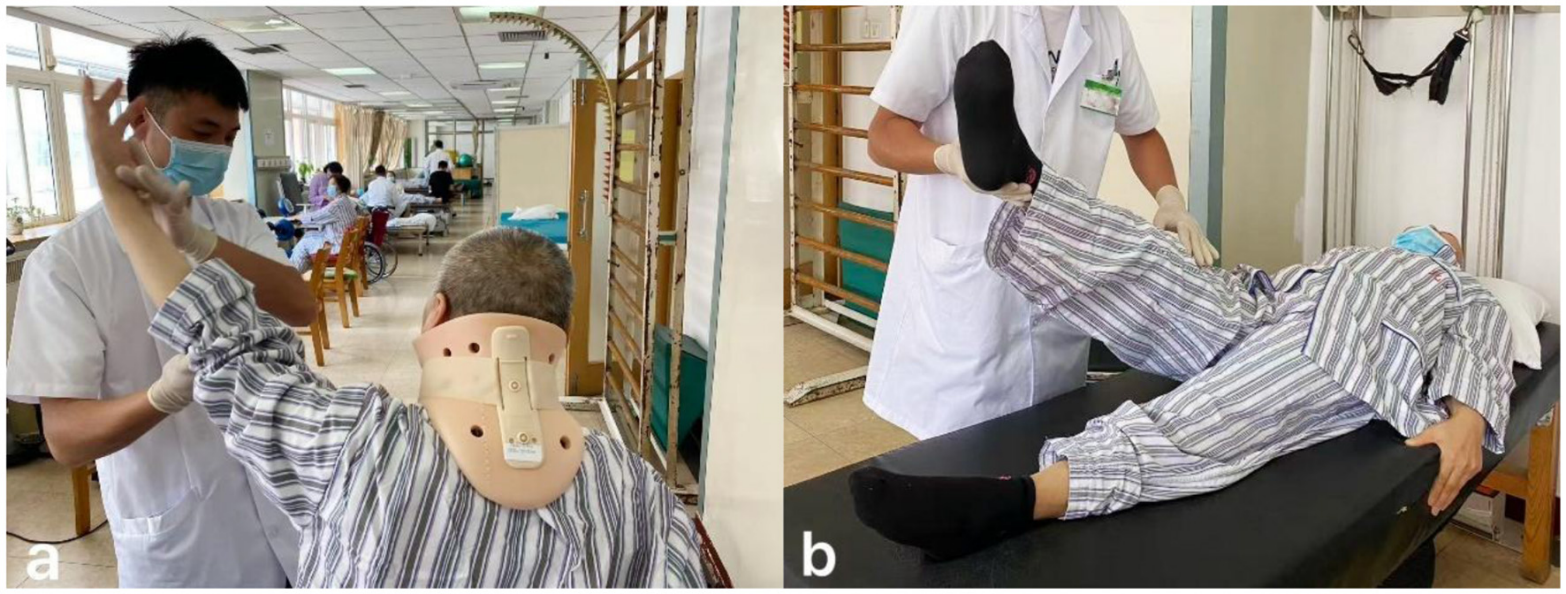
**(a)** Upper limb motor function training. **(b)** Lower limb motor function training.

**Figure 2:**
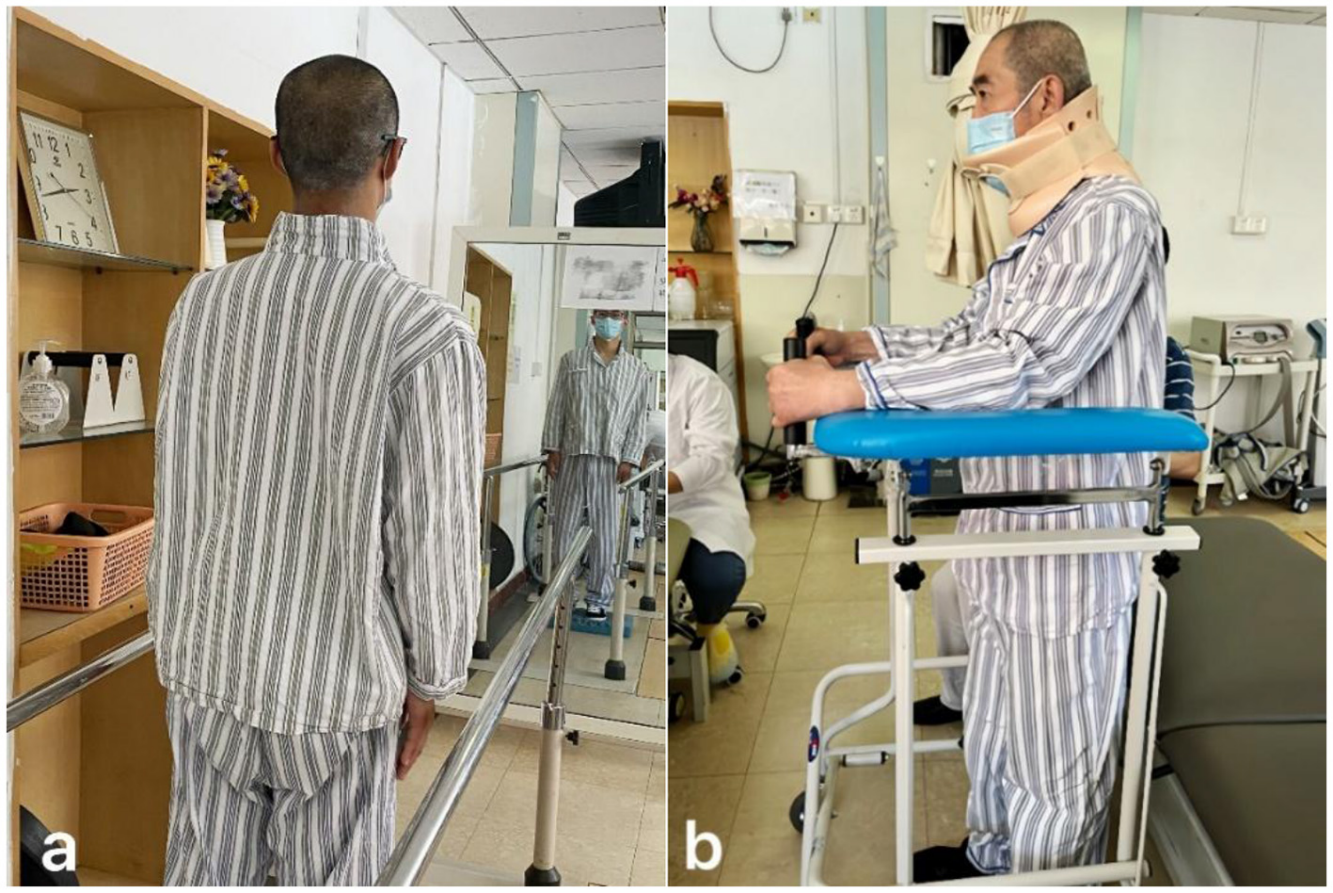
**(a)** Visual feedback-assisted standing balance training. **(b)** Walking aid-assisted standing balance training.

**Figure 3:**
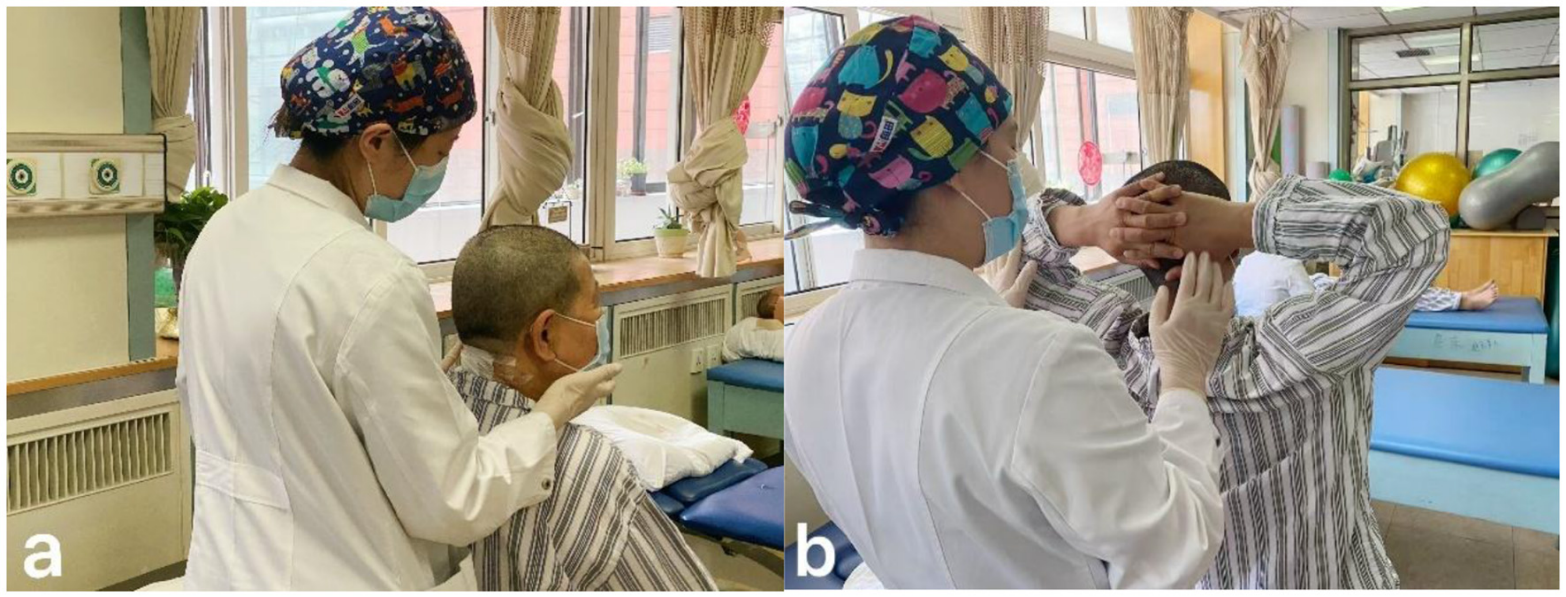
**(a)** Active cervical rotation range of motion training. **(b)** Cervical extension resistance isometric muscle strength training.

Patients received rehabilitation treatment in hospital for 7 days under the guidance of a professional team composed of rehabilitation doctors, physiotherapists, occupational therapists, and nurses. The treatment was based on Chinese expert consensus on the implementation of enhanced recovery after surgery in posterior cervical spine surgery (22). From the third day after surgery, the training was conducted for approximately 60 min each time, twice a day, and based on the patients' functional performance to adjust the exercise prescription. The nerve function training of the limbs and hands includes proprioceptive neuromuscular facilitation practices, muscle strength training and hand function training, up to 30 min at a time (Figure 1). Training on transfers, balance and gait up to 20 min at a time (Figure 2). If the neck pain was mild, perform cervical function training includes cervical range of motion training and isometric muscle strength training, the training lasts no more than 20 min (Figure 3). After the 7-day rehabilitation treatment was completed, we provided patients with a home-based rehabilitation programme that includes cervical spine function training, according to their functional impairments. Patients were discharged from the hospital and started a daily rehabilitation exercise at home.

### 2.4 Data collection

**Figure 4:**
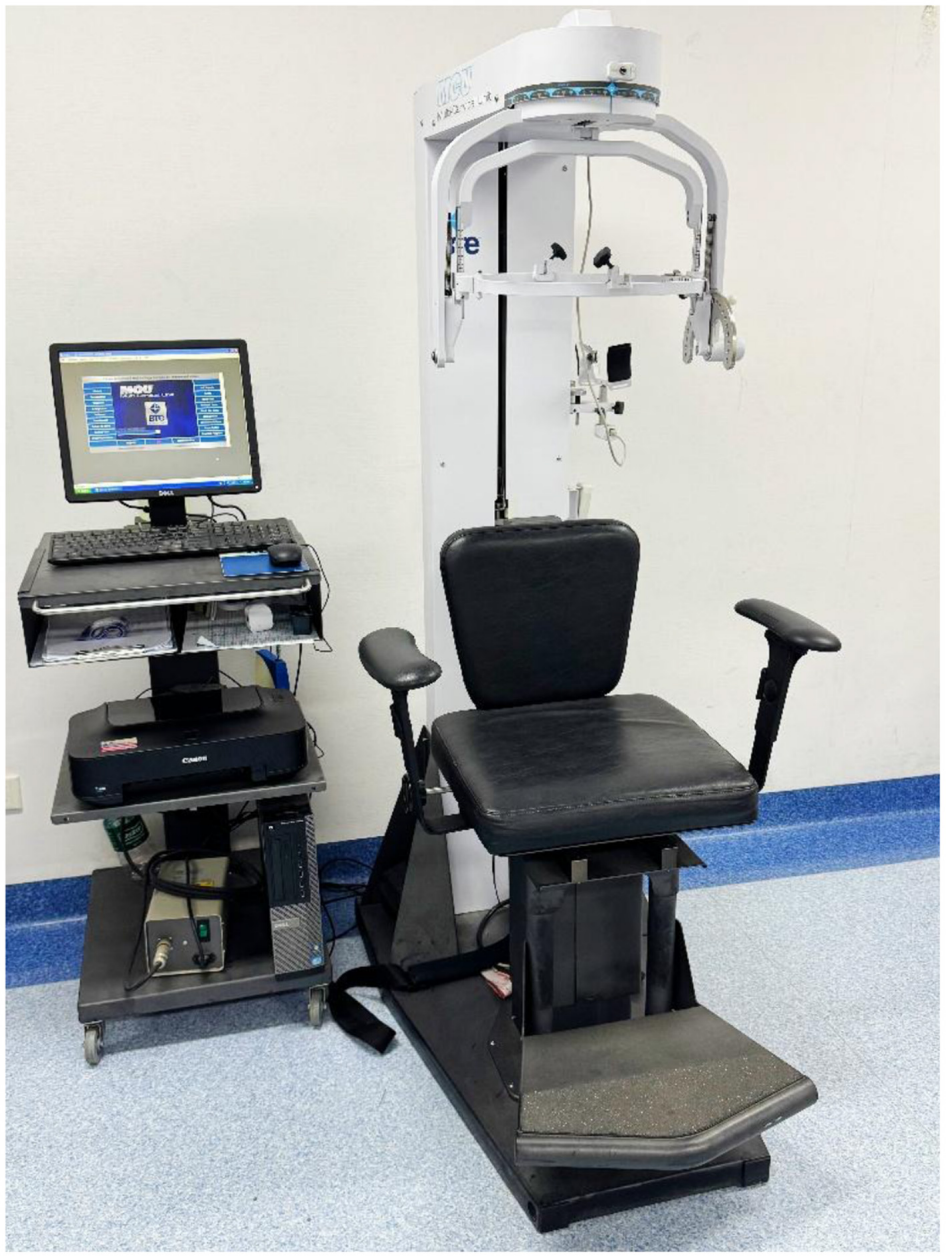
The Multi-Cervical Unit (MCU, BTE Technologies, Inc, Hanover, MD) is a biomechanical system designed to measuring the active range of motion of the neck and the isometric strength of neck muscles. The unit is equipped with an armchair that rotates 90° for measurement of lateral flexions, with adjustable seat height, lumbar support and armrests, and a shoulder restraint system to secure the subject within the seat in order to isolate the cervical spine during testing. It also contains a unique head assembly system (movable inner and outer head brace) designed to cause the subject's head to move safely in different planes.

**Figure 5:**
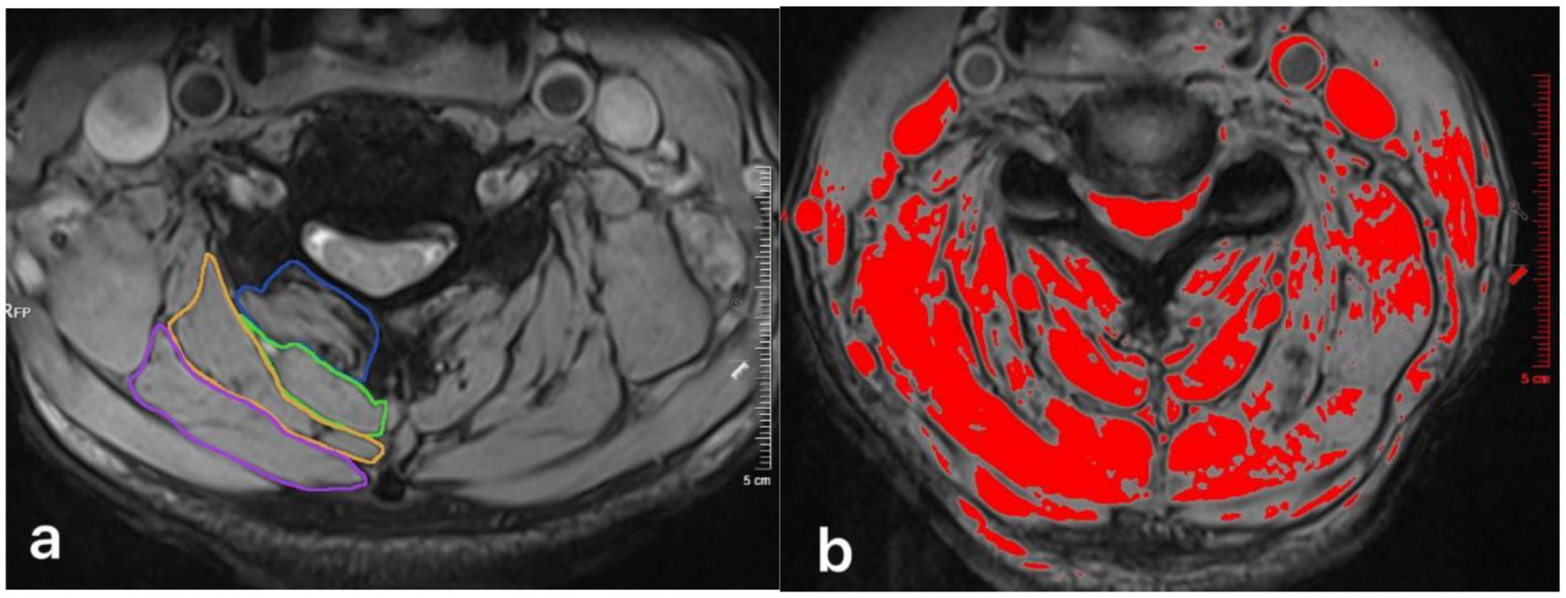
**(a)** Measurement of pre-operatively total CSA at C4/5 level for the multifidus (MF, blue circle), semispinalis cervicis (SC, green circle), semispinalis capitis (SCap, orange circle), and splenius capitis (SpC, pink circle). **(b)** Thresholding technique was used to mark the pre-operative FI and calculate the fCSA.

Information was extracted from the hospital's electronic medical record system and the collected patient follow-up case report forms. The data collected included: (1) basic information: gender, age, surgical segment, longest follow-up time; (2) visual analog scale (VAS) score: a total score of 10 points, with a higher score indicating more severe pain; (3) modified Japanese orthopedic association (mJOA) score: a total score of 17 points, with a higher score indicating better neurological function. This is currently a widely used evaluation index for cervical spinal cord function (23); (4) neck disability index (NDI): a total score of 50 points, with a higher score indicating worse cervical spine function, and its reliability and validity have been effectively verified (24); (5) cervical range of motion (ROM) and muscle strength: using Multi-Cervical Unit (MCU, BTE Technologies, Inc, Hanover, MD) for cervical flexion and extension ROM and isometric muscle strength tests (25, 26), recording cervical flexion ROM, extension ROM, flexion strength and extension strength (Figure 4); (6) functional cross-sectional area (fCSA) of posterior cervical muscles: patients underwent 3.0T magnetic resonance imaging (MRI, GE Signa HDxt 3.0T) before surgery, and the images were stored in the hospital's Picture Archiving and Communication System (PACS, United Imaging Medical Technology Co., Ltd., Shanghai, China). The intervertebral disc cross-sectional T2-weighted images of the C3/4, C4/5, C5/6, and C6/7 segments were downloaded to the computer, and two physicians simultaneously used the ImageJ system (Version 1.54p, National Institutes of Health, Bethesda, Maryland) for measurement, and took the average value. Four areas within the cross-sectional muscle were selected and the signal values were measured. The maximum value among them was selected as the threshold to distinguish normal muscle tissue from fat tissue. The cross-sectional areas (CSA) of the multifidus (MF), semispinalis cervicis (SC), semispinalis capitis (SCap), and splenius capitis (SpC) on both sides were obtained, and the fatty infiltration (FI) was calculated based on the measured muscle threshold, and the fCSA was calculated as fCSA = CSA × (1-FI) (27) (Figure 5).

This study collected the VAS scores, mJOA scores and NDI scores of the patients before and 3 months after laminoplasty. It also conducted a correlation analysis between pre-operative cervical spine function and post-rehabilitative functional scores.

### 2.5 Statistical analysis

All the statistical analyses were performed using SPSS version 27.0 (IBM Corp., Armonk, NY, United States). The Shapiro–Wilk test was used to assess the normality of the data distribution. Normally distributed continuous variables are presented as the mean ± standard deviation (SD) and non-normally distributed continuous variables are expressed as medians with ranges. Categorical variables are reported as frequencies and percentages [*n* (%)]. Paired sample *t*-test was used to compare pre-operative and post-operative index. The Pearson correlation coefficients were calculated and multivariate linear regression analysis was used to assess the linear correlation between pre-operative and post-operative target, with a significance level set at *p* ≤ 0.05.

## 3 Results

### 3.1 Clinical features

A total of 30 patients who underwent laminoplasty and received inpatient rehabilitation treatment were included in this study. No serious complications such as nerve injury, post-operative infection, or poor wound healing occurred in any of the patients during the follow-up period. The average age was 59.70 ± 8.41 years, including 17 (56.7%) male and 13 (43.3%) female. Among them, 20 (66.7%) underwent C3-C7 segment open-door laminoplasty, and 10 (33.3%) underwent non- C3-C7 segment open-door laminoplasty. Within the group there was no significant difference in the VAS scores before and 3 months after surgery (*p* > 0.05), while there were significant differences in the mJOA scores and NDI scores within the group before and 3 months after surgery (*p* ≤ 0.05). The average pre-operative cervical flexion ROM was 36.7° ± 9.5°, the extension ROM was 37.8° ± 9.5°, and the flexion muscle strength was 8.1 ± 4.3 lbs, the extension muscle strength was 11.0 ± 5.8 lbs (Table 1).

**Table 1:** Baseline characteristics and clinical features of patients.

**Variables**	**Total**		
Age; mean+SD	59.70 ± 8.41		
Gender			
Male; *n* (%)	17 (56.7)		
Female; *n* (%)	13 (43.3)		
Surgical segment; *n* (%)			
C3-C7	20 (66.7)		
Non C3-C7	10 (33.3)		
Obsevation index	Pre-operation	Post-operation	*p*-Value
VAS; mean + SD	2.6 ± 2.2	3.2 ± 2.3	0.262
mJOA; mean + SD	13.4 ± 2.1	14.6 ± 2.0	**0.008**
NDI; mean + SD	7.2 ± 7.2	10.1 ± 6.9	**0.050**
Flexion ROM; mean + SD, degrees	36.7 ± 9.5		
Extension ROM; mean + SD, degrees	37.8 ± 9.5		
Flexion strength; mean + SD, lbs	8.1 ± 4.3		
Extension strength; mean + SD, lbs	11.0 ± 5.8		

Statistically significant values are in bold (p ≤ 0.05).

Table 2 presented the measurement values and sum values of posterior cervical muscles' fCSA on both sides of each cervical segment before surgery. The measurement of posterior cervical muscles included MF, SC, Scap, and SpC.

**Table 2:** Pre-operative posterior cervical muscles fCSA index.

**Surgical segment**	**Cervical muscle**	**Side**	**C3/4**	**C4/5**	**C5/6**	**C6/7**	**Sum**
fCSA, cm^2^	MF	Left	1.22 ± 0.39	1.26 ± 0.44	1.27 ± 5.83	1.57 ± 0.59	5.33 ± 1.66
		Right	1.29 ± 0.38	1.20 ± 0.46	1.15 ± 0.44	1.50 ± 0.44	5.11 ± 1.38
	SC	Left	0.99 ± 0.44	0.88 ± 0.52	0.72 ± 0.38	0.59 ± 0.30	3.17 ± 1.17
		Right	1.05 ± 0.42	0.78 ± 0.42	0.59 ± 0.34	0.62 ± 0.31	3.03 ± 1.07
	Scap	Left	1.97 ± 1.19	1.80 ± 1.01	1.32 ± 0.79	1.31 ± 0.68	6.40 ± 3.18
		Right	1.54 ± 1.07	1.53 ± 1.14	1.06 ± 0.66	1.13 ± 0.67	5.25 ± 3.17
	SpC	Left	1.39 ± 1.17	1.64 ± 1.24	1.74 ± 1.20	1.53 ± 1.17	6.30 ± 3.94
		Right	1.01 ± 0.70	1.12 ± 0.83	1.37 ± 1.07	1.21 ± 0.84	4.70 ± 2.94

Sum, calculate the sum value of posterior cervical muscles from segments C3 to C7.

### 3.2 Correlation analysis

The correlation analysis showed that there was no correlation between the pre-operative cervical flexion ROM, extension ROM, flexion muscle strength and the post-operative 3-month observation indicators (*p* > 0.05). The pre-operative cervical extension muscle strength was weakly positively correlated with the post-operative VAS score (*r* = 0.364, *p* = 0.048) and was moderately positively correlated with the NDI score (*r* = 0.448, *p* = 0.013), however there was no correlation with the mJOA score (*p* > 0.05; Table 3).

**Table 3:** Correlation analysis of post-operative observation index and pre-operative cervical spine function.

**Observation index**	**VAS**		**mJOA**		**NDI**	
	***r*** **-value**	***p*** **-Value**	***r*** **-value**	***p*** **-Value**	***r*** **-value**	***p*** **-Value**
Flexion ROM	0.001	0.996	−0.141	0.457	0.160	0.397
Extension ROM	0.259	0.167	0.183	0.332	0.111	0.561
Flexion strength	0.144	0.448	0.008	0.966	0.214	0.256
Extension strength	0.364	**0.048**	−0.111	0.558	0.448	**0.013**

Statistically significant values are in bold (p ≤ 0.05).

To further verify whether extension strength was an independent predictor of VAS score and NDI score, we conducted a multiple linear regression analysis. Under the condition of controlling for flexion ROM, extension ROM, and flexion strength, we evaluated the independent impact of extension strength on post-operative indicators. The regression analysis results showed that extension strength had a significant and positive independent predictive effect on post-operative VAS score (β = 0.256, *p* = 0.025) and NDI score (β = 0.789, *p* = 0.024), indicating that extension strength was a potential independent predictor of VAS score and NDI score after surgery (Table 4).

**Table 4:** Multiple linear regression analysis of pre-operative cervical spine function predicting post-operative observation index.

**Observation index**	**Variant**	**SE**	**β (95%CI)**	***t*-value**	***p*-Value**
VAS	Constant	1.810			
	Flexion ROM	0.054	−0.093 (−0.204, 0.018)	−1.733	0.095
	Extension ROM	0.051	0.083 (−0.022, 0.187)	1.621	0.118
	Flexion strength	0.137	−0.157 (−0.440, 0.126)	−1.142	0.264
	Extension strength	0.107	0.256 (0.036, 0.476)	2.392	**0.025**
NDI	Constant	5.538			
	Flexion ROM	0.165	−0.033 (−0.373, 0,306)	−0.203	0.841
	Extension ROM	0.156	−0.016 (−0.337,0.305)	−0.100	0.921
	Flexion strength	0.42	−0.397 (−1.263, 0.469)	−0.944	0.354
	Extension strength	0.327	0.789 (0.114, 1.463)	2.409	**0.024**

SE, Standard error; CI, Confidence interval.

Statistically significant values are in bold (p ≤ 0.05).

Table 5 presented the correlation analysis results between the pre-operative sum values of posterior cervical muscles' fCSA on the left and right sides and the post-operative observation indicators. The results showed that for the sum of posterior cervical muscles, pre-operative fCSA of the right MF was weakly negatively correlated with the post-operative NDI score (*r* = −0.369, *p* = 0.045). The fCSA of other posterior cervical muscles did not show a correlation with the post-operative 3-month observation indicators (*p* > 0.05).

**Table 5:** Correlation analysis of post-operative observation index and pre-operative cervical spine fCSA index.

**Observation index**	**Cervical muscle**	**Side**	**VAS**		**mJOA**		**NDI**	
			***r*** **-Value**	***p*** **-Value**	***r*** **-value**	***p*** **-Value**	***r*** **-Value**	***p*** **-Value**
fCSA-Sum	MF	Left	−0.144	0.448	0.021	0.910	−0.300	0.107
		Right	−0.103	0.588	0.105	0.582	−0.369	**0.045**
	SC	Left	−0.039	0.837	−0.161	0.394	−0.146	0.442
		Right	0.045	0.813	−0.338	0.067	−0.124	0.515
	Scap	Left	−0.108	0.570	−0.156	0.410	−0.124	0.514
		Right	−0.152	0.423	−0.212	0.261	−0.101	0.595
	SpC	Left	−0.025	0.896	−0.188	0.319	−0.135	0.475
		Right	−0.052	0.786	−0.109	0.565	0.063	0.741

Sum, calculate the sum value of posterior cervical muscles from segments C3 to C7. Statistically significant values are in bold (p ≤ 0.05).

## 4 Discussion

The laminoplasty is one of the standard surgical procedures for treatment of multilevel DCM, its clinical efficacy has been widely recognized. However, due to the dissection of muscles during the exposure of the surgical area, posterior cervical surgery has a significant negative impact on the posterior cervical muscles. In this study, all patients underwent single-door laminoplasty with the preservation of the right musculo-ligamentous complex. Previous studies have confirmed that this surgical method has long-term stable efficacy (28), and the cervical curvature can be maintained well. However, there are still problems such as decreased cervical mobility and atrophy of the posterior cervical muscles, which are difficulties in the post-operative rehabilitation of patients (27). To solve this difficult clinical problem, our research team, based on conventional rehabilitation treatment, added early post-operative rehabilitation training targeting cervical function, including active cervical ROM exercises and isometric muscle strength exercises of the neck. However, the above treatments did not show the advantages in rapidly relieving post-operative axial symptoms in the clinical follow-up (18).

This study appeared to indicate that cervical extension strength is a potential independent predictor of VAS score and NDI score after surgery. However, the result suggested that the better the pre-operative cervical extension strength, the more severe the axial symptoms would be after laminoplasty. It is contrary to conventional understanding and previous research trends. Through further literatures review, we found that previous researchers' exploration of cervical spine function was often based on analysis of surgical-related structural indicators such as changes in cervical curvature (29–31). Better cervical spine function can lead to a more stable post-operative structure and result in better objective measurement indicators. However, post-operative neck pain is a subjective sensation for patients and often has no direct correlation with objective measurement indicators, and is influenced by various factors (16). Beneck et al. (32) believed that in the early post-operative period, the accumulation of lactic acid in muscle tissue will stimulate the nerve endings, thereby causing local muscle pain. Damaged soft tissues and impaired blood circulation both lead to the accumulation of inflammatory mediators in the surgical area. This local accumulation stimulates the peripheral terminals of primary sensory neurons and increases sensitivity to pain, resulting in obvious pain manifestations (33). In addition, atrophied neck muscles have a situation of denervation (34), patients with poor cervical extension strength before surgery may experience less post-operative pain than others. Uehara et al. (17) discovered that change in the VAS of axial pain from before surgery to 3 months after surgery showed a greater decreased neck extension muscle strength resulting in severer axial pain. Therefore, considering the damage that laminoplasty may cause to posterior cervical muscle of the patients, it was observed that patients with better pre-operative cervical extension strength showed a significant decrease in muscle strength after surgery. The accumulation of inflammatory substances stimulates nerve endings, which may lead to more obvious axial symptoms. It is undeniable that the number of cases included in this study is limited. Therefore, the result of this study need to be further validated and analyzed through prospective large-sample studies.

Based on the results of MRI, correlation analysis showed that for the sum of posterior cervical muscles, pre-operative fCSA of the right MF was weakly negatively correlated with the post-operative NDI score, but we did not find a correlation with other observation indicators. Therefore, our study did not indicate a correlation between pre-operative fCSA and post-operative functional indicators. Naghdi et al. (20) conducted a correlation analysis on the pre-operative cervical spine MRI measurement values and post-operative indicators of 171 patients, and discovered the predictive value of the pre-operative morphology characteristics of cervical posterior muscles for post-operative function. Wong et al. (35) revealed that the pre-operative asymmetry of cervical muscles from C5 to C7 segments was a risk factor for adjacent segment disease after anterior cervical discectomy and fusion. The above studies confirmed the predictive value of pre-operative measurements of the posterior cervical muscles, therefore further prospective study can be designed to increase imaging measurement indicators of cervical posterior muscles, in order to explore their impact on post-operative function.

This study is the first to explore correlation between pre-operative cervical spine function and post-rehabilitation function after laminoplasty. The result appeared to indicate that cervical extension strength is a potential independent predictor of post-operative axial symptoms. For patients with stronger cervical extension strength, their post-operative pain can be quite obvious due to more pronounced muscle weakness and sensitization of pain perception. This might be a problem that we had overlooked before. Therefore, pain management after surgery is crucial for early rehabilitation exercises. Nie et al. (19) discovered that for patients with post-operative axial symptoms after cervical surgery, both peripheral and central pain sensitization exist. Compared with cervical range of motion training, progressive resistance cervical muscle strength training can reduce pain sensitization and reduce the occurrence of post-operative axial symptoms. Their study suggests that we need to further refine our existing post-operative cervical spine function training program, focusing on cervical muscle strength training as the key component of functional exercises, which might effectively alleviate post-operative pain. Additionally, we can also apply physical agents therapy such as transcutaneous electrical nerve stimulation (36), low-frequency ultrasound therapy (37), and high-intensity laser therapy (38) to relieve post-operative pain. Our study offered a new perspective for improving the axial symptoms of patients after laminoplasty. However, as it was a retrospective study with a limited sample size, our research results still require further experimental verification.

## 5 Conclusion

Our result suggested that stronger pre-operative cervical extension strength may be important predictors of post-operative functional rehabilitation in patients after laminoplasty, especially in terms of axial symptoms.

## 6 Limitations

This study is a retrospective study, which has several limitations such as numerous confounding factors, incomplete data, and resulting in a relatively small sample size. The single-center design and limited sample size may have reduced statistical power, potentially obscuring the significance of certain clinical indicators. In addition, the collection of the observation indicators in this study was mainly focused on 3 months after the surgery. Further, collection of long-term post-operative indicators is required.

## Data Availability

The raw data supporting the conclusions of this article will be made available by the authors, without undue reservation.
